# Schwannoma of the arythenoid

**DOI:** 10.1590/S1679-45082013000200015

**Published:** 2013

**Authors:** Carlos Eduardo Molinari Nardi, Alexandre Wakil Burzichelli, Elio Gilberto Pfuetzenreiter, Rogerio Aparecido Dedivitis

**Affiliations:** 1 Núcleo de Cirurgia de Cabeça e Pescoço de Santos, Santos, SP, Brazil; 2 Hospital da Irmandade da Santa Casa da Misericórdia de Santos, Santos, SP, Brazil

**Keywords:** Neurilemmoma, Laryngeal neoplasms, Arythenoid cartilage, Case reports

## Abstract

Schwannoma is a benign encapsulated tumor that originates from the Schwann cells lining nerve fibers outside the central nervous system. We report a rare case of schwannoma that arose from the left arythenoid cartilage The patient underwent excision of the mass through microlaryngeal endoscopic procedure. No recurrence was observed during follow-up.

## RESUMO

O schwanoma é um tumor benigno encapsulado, que tem origem nas células de Schwann que revestem as fibras nervosas, fora do sistema nervoso central. É relatado aqui um caso raro de um schwanoma que surgiu da aritenoide esquerda. A paciente foi submetida à excisão da massa por meio de procedimento de endoscopia microlaríngea. Nenhuma recorrência foi observada durante o seguimento.

## INTRODUCTION

A schwannoma is a benign encapsulated tumor that originates from the Schwann cells lining nerve fibers outside the central nervous system^(1)^. It is significantly more likely to affect sensory nerves than motor nerves. It is typically slow-growing, well-circumscribed, and located on the proximal nerves or spinal nerve roots^(2)^. It is usually a solitary lesion, but may be multiple and even associated with von Recklinghausen disease, which can be considered as disseminated neurofibromatosis^(3)^. Neurilemmomas equally affect both genders, and they occur most often during the fifth and sixth decades of life^(2)^. The laryngeal symptoms are related to the mass effect of the slow-growing lesion and include throat pain, odynophagia, dysphagia, stridor, dyspnea, hoarseness, and sensation of a lump in the throat^(4)^.

We report a case of arythenoid schwannoma.

## CASE REPORT

**Figure 1:**
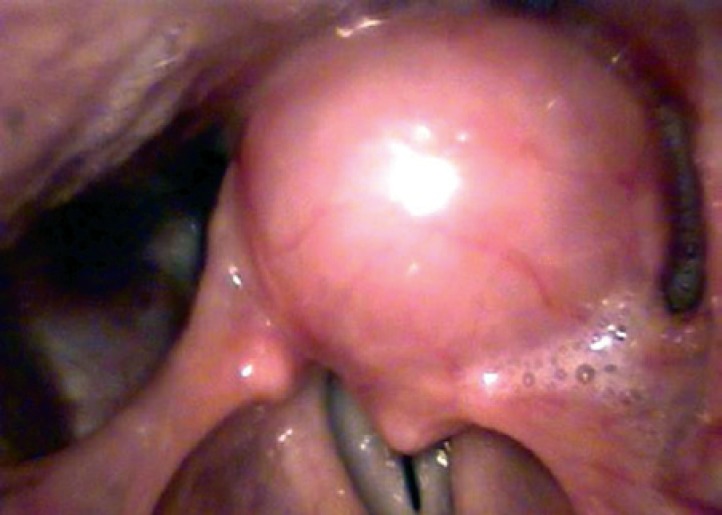
Laryngoscopic aspect of the mass situated in the left arythenoid

**Figure 2:**
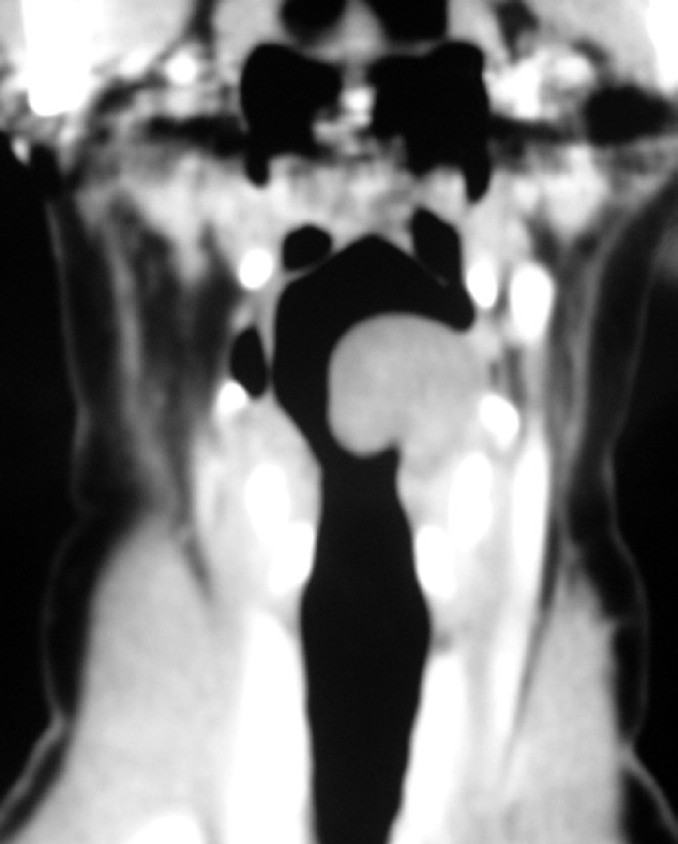
Computed tomography scan showing a well-defined homogeneous mass in the left arythenoid

**Figure 3:**
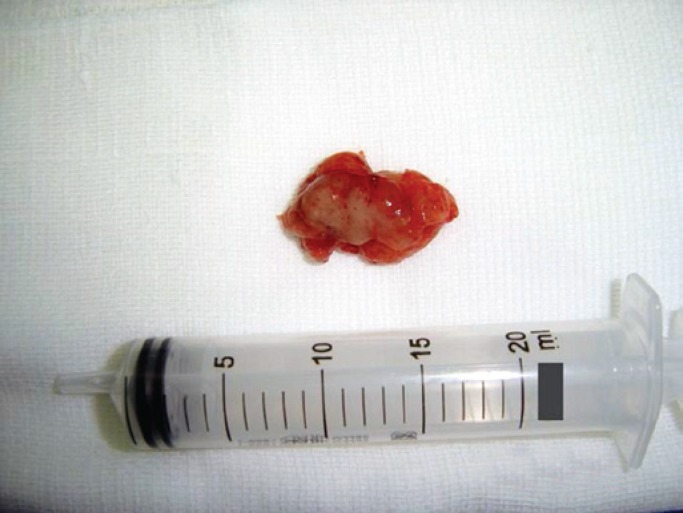
Specimen of complete tumor resection

A 44 year-old female patient, teacher, presented with dysphonia for three months. She underwent laryngoscopic examination with visualization of a regular and apparently well-defined mass in the left arythenoid (Figure 1). The computed tomography scan showed a solid lesion, with no central necrosis, limited to the left arythenoid (Figure 2). Microsurgery of the larynx with complete resection of the lesion was performed. Gross examination revealed a brownish, firm, and elastic tissue measuring 3.0×1.8×0.6cm. The cut surface showed a soft and heterogeneous light-brown tissue. Benign schwannoma was diagnosed (Figure 3). The patient was reassessed at 6 months of follow-up, showing normal laryngoscopy. The Ethics Committee of the *Irmandade da Santa Casa da Misericórdia de Santos* approved the study under number 54/10.

## DISCUSSION

In a review of 722 cases of benign tumors of the larynx, New and Erich^(5)^ first reported one case of schwannoma. Since then, sporadic reports of laryngeal involvement have been found in the literature.

Laryngeal schwannomas are distinctly rare, although those of the head and neck region account for about half of the recorded cases at all sites, the most common site being the parapharyngeal space^(1)^. These tumors mostly involve the aryepiglottic folds (80%). The most common origin is the internal branch of the superior laryngeal nerve after its penetration through the thyrohyoid membrane^(4)^. Confusion sometimes exists between a neuroma and a neurofibroma. A neurofibroma is not specifically encapsulated and is characterized by the proliferation of lining cells and nerve fibres^(3)^.

Laryngeal cysts, internal laryngoceles and neurinomas associated with Recklinghausen' disease have to be considered in the differential diagnosis. A computed tomography scan is valuable to show the extension of the mass and the typical image of a heterogeneous densitometric enhancement of large schwannomas^(4)^.

The diagnosis can be made by taking either a fine-needle aspiration or incisional biopsy of the mass via the endolaryngeal route^(1)^. Immunocytochemical staining for S-100 protein is used to identify tumors of Schwann cell origin^(3)^. Three criteria are necessary to establish a histopathologic diagnosis: presence of a capsule, identification of Antoni A and B areas, and positivity of tumor cells for S-100 protein^(2)^. Besides the use of computed tomography, magnetic resonance imaging is a valuable technique for delineating the anatomical extent of the lesion^(1)^.

Because of the radioresistant nature of the Schwannoma, radiation therapy is not indicated. Surgery is the treatment of choice^(4)^. Ideally, a neurilemmoma should be totally excised, but anatomic constrains sometimes make this difficult. The preferred method is microlaryngeal endoscopic excision with either conventional microlaryngoscopy instruments or a CO_2_ laser. An open approach may be necessary for larger lesions^(2)^, such as medial thyrotomy, lateral pharyngotomy, and external lateral thyrotomy excising the upper half of the thyroid lamina^(4)^. In these cases, a prior tracheostomy is mandatory^(1)^. The approach for each individual case should be planned according to the site, extent, and presentation of the tumor^(4)^.

In this case, the patient had no vocal complaints or dysphagia and the diagnosis was obtained by examination findings. The CT scan was helpful in showing lesions limited to the arythenoid, which could be resected by microsurgery of the larynx. Good outcome was observed in the postoperative period and follow-up.
